# Evaluating plasma and tissue biopsy for DNA methylation markers in early colorectal cancer detection: a systematic review

**DOI:** 10.2478/abm-2025-0029

**Published:** 2025-10-31

**Authors:** Hans Ezekiel T. Olorosisimo, Charlemagne G. Sumperos, Aeryll Lesley A. Adviento, Angel Ann T. Lachica, John Louie A. Cabalteja, An Gheline R. Ubiña, Josiah T. Valentin, Pamela Rose Bremner

**Affiliations:** Far Eastern University Manila, Institute of Health Sciences and Nursing, Department of Medical Technology, Manila, 1015, Philippines olorosisimohansezekiel@gmail.com

**Keywords:** blood plasma, colorectal cancer, DNA methylation markers, sensitivity, specificity, tissue biopsy

## Abstract

**Background:**

DNA methylation markers are emerging as promising diagnostic tools for the early detection of colorectal cancer (CRC) that can significantly improve survival rates.

**Objective:**

To compare the capabilities of blood plasma and tissue biopsy for detecting these markers in early CRC stages by diagnostic measures.

**Methods:**

Nine studies published from 2020 to 2024 were analyzed, and the study quality was assessed using the Quality Assessment of Diagnostic Accuracy Studies-2 (QUADAS-2) tool.

**Results:**

This review reaffirms tissue-based samples as the gold standard based on the superior sensitivity and specificity with markers such as SFMBT2 being over 90% in the 2 parameters. However, due to its invasive nature, it challenges applicability for asymptomatic patients or routine screening. Plasma-based markers (SEPT9 and HAND1) offer a noninvasive alternative, with moderate sensitivity (40%–75.8%) and high specificity (69%–94.7%), while combining multiple markers improves overall diagnostic performance. However, most plasma-based assays evaluated in this review do not yet meet the 2021 Centers for Medicare and Medicaid Services approval benchmarks of ≥74% sensitivity and ≥90% specificity compared with colonoscopy, which shows the need for further optimization before its clinical implementation. QUADAS-2 illustrated a potential high risk of bias in patient selection and flow/timing domains, which underscores the need for more standardized diagnostic workflows and assay protocols.

**Conclusions:**

Future research should focus on multi-marker panels, adherence to regulatory thresholds, cost-effectiveness analyses, and clear clinical management pathways to facilitate the widespread implementation of plasma-based CRC screening.

Colorectal cancer (CRC), which includes cancer of the colon and the rectum, is a major health concern, being the third most frequently diagnosed and the second leading cause of cancer-related deaths worldwide [1]. Early detection is key to increasing survival rates and patient outcomes. Another major area of recent CRC diagnostics advancement that has gained attention is DNA methylation markers and their potential for identifying early-stage disease. Aberrant methylation patterns on specific genes represent an epigenetic modification, where methyl groups are added to cytosine-phosphate-guanine (CpG) sites. These changes play a critical role in gene regulation but are also implicated in cancer development, making them valuable biomarkers for early detection, prognosis, and treatment monitoring [2]. These markers are detectable through various diagnostic methods. Identifying these markers includes tissue biopsies, usually taken via colonoscopy, considered as the gold standard for cancer marker identification [3]. However, this is invasive, uncomfortable, and unsuitable for repeated monitoring in asymptomatic people. Liquid biopsies, like blood plasma testing, are much less invasive. Circulating tumor DNA (ctDNA) blood plasma tests generally provide access to more genetic and epigenetic alterations broadly reflective of systemic tumor activity [4]. The utility of the widely used CRC methylation marker SEPT9, which has shown high sensitivity and moderate specificity, may serve as an alternative screening method for individuals who decline the fecal immunochemical test (FIT) for occult blood [5]. Other studies suggest that combining methylation markers may improve the performance of CRC detection in specific populations [6]. In line with this, Yuan et al. [7] demonstrated that SEPT9, when combined with SDC2, exhibits greater reliability compared with other methods. However, despite their potential, the use of plasma-based markers in routine clinical settings remains uncertain, as their sensitivity and specificity for early CRC detection are still under evaluation. This was supported by the findings of Kassid et al. [8], which showed that the levels of SEPT9 markers were significantly higher in colon cancer tissues than in patients’ serum samples.

Despite the latest technological advances in diagnosis and treatment, CRC remains a major global health problem. Therefore, early detection significantly affects patient outcomes [9]. Advances in DNA methylation markers have had great potential for early CRC detection over the past decade. Thus, controversies about the most appropriate method to detect these markers have reappeared, with blood plasma and tissue biopsy as leads. Although invasive and uncomfortable for patients, tissue biopsies or colonoscopies are considered as the gold standard for identifying cancer markers [10]. According to Laugsand et al. [11], the tissue biopsy method is unfit for repeated monitoring and detecting early markers in asymptomatic patients. However, its invasiveness is of least concern for liquid biopsies like blood plasma. It gives a more comprehensive picture of cancer markers and can enhance early detection, prompting better disease monitoring. Despite the proliferation of candidate methylation markers, there is no consensus that exists on an optimal panel due to heterogeneity in study designs and cohort populations [12]. Additionally, preanalytical and analytical protocols (e.g., DNA extraction, bisulfite conversion) vary widely across labs, which further complicates translation in the clinical setting. In 2021, regulatory bodies like the United States Preventive Services Task Force (USPSTF) highlighted this lack of standardization as a barrier to adopting plasma-based methylation tests for CRC screening [13]. To fill this gap, this systematic review aims to compare the diagnostic efficacy of blood plasma versus tissue biopsy for identifying DNA methylation markers for early CRC diagnosis and will offer evidence-based insights as guidance for clinical decision-making and research aims.

Furthermore, the general objective of this study is to critically evaluate the effectiveness of utilizing blood samples, specifically plasma and tissue biopsy specimens, in detecting DNA methylation markers for the early diagnosis of CRC. This study aims to determine the most effective and reliable DNA methylation markers by comparing their diagnostic function and performance for the early detection of CRC, including specificity and sensitivity, in blood and tissue biopsy. Additionally, the study seeks to assess the practicality and feasibility of the methods in detecting DNA methylation markers from blood plasma and tissue biopsy in a clinical setting.

Early detection is crucial in CRC survival rates and outcomes. Specifically, this systematic review on comparing blood plasma and tissue biopsy for the detection of DNA methylation markers for the early diagnosis of CRC would be beneficial in the following fields:

(1) In the molecular biology practice, this study will provide molecular biologists with a better perspective of the effectiveness of detecting DNA methylation markers in blood plasma and tissue biopsy as a method for early diagnosis. This review will aid in understanding the efficient detection of early-stage cancers. This will also provide more viable options in detection for improving laboratory procedures;
(2) In molecular biology education, where students, researchers, and professionals will better understand the optimal option for the specimen of use for early detection and diagnosis of cancer. This will allow them to test suspected cancer patients easily;
(3) In health research, as this study will pave the way to new research directions for early detection of different types of cancers. For researchers and molecular biologists who are finding ways to treat cancer, the results of this systematic review can be an important source of information. This study will help in the development of new methods for better accuracy and precision in diagnosing the early stages of cancer by determining the limitations of the present methods;
(4) Public health will be significantly aided by this systematic review, especially when it comes to the early detection and diagnosis of CRC. The study results could raise awareness among the general public about the methods for early detection and their effectiveness. This could give an option for patients who are suspected to have the condition and would want to undergo tests to have a better quality of life and less possible expenses for the treatment of CRC.

## Methods

### Literature search

The researchers used different literature databases to collect research materials for this paper review, such as PubMed Center (PMC), National Center for Biotechnology Information (NCBI), Scopus, and Cochrane Library. The research papers and journals collected from these databases were accessed, and the data gathered were verified in November 2024. Key terms were used to find the related literature such as DNA methylation marker OR DNA methylation, blood plasma OR plasma OR blood sample OR serum OR blood serum, tissue biopsy OR tissue sample OR tissue, early detection OR early diagnosis, and CRC OR early CRC.

### Eligibility criteria

Comparing blood plasma and tissue biopsy for DNA methylation marker detection in early colorectal diagnosis via a systematic review includes papers and journals that meet the following criteria: (1) published materials from literature databases, that is, PMC NCBI, Scopus, and Cochrane Library; (2) researches published in English; (3) papers that are accessible publicly in full-text; (4) literature published in 2020 to the present year; (5) reports on DNA methylation markers relevant to CRC; (6) early CRC and stages indicated (Stages 0–II); (7) studies that assess DNA methylation markers in blood plasma and/or tissue biopsy samples; and (8) diagnostic performance metrics are reported such as sensitivity and specificity. However, studies that were excluded in this review paper were seen applicable in one of the following criteria: (1) research and journals that were published from other non-credible search engine sites aside from the aforementioned databases; (2) papers that were published in a language other than English; (3) published researches produced earlier than 2020; (4) literature with a research design of either a systematic review or meta-analysis; (5) studies without reference to DNA methylation markers or with markers not validated for CRC diagnosis; (6) researches with CRC stage not indicated or focusing on early CRC (Stages III–IV); (7) studies that use only stool samples, urine, or non-tissue/plasma matrices; and (8) papers that are lacking diagnostic metrics.

### Tools and selection strategy

The researchers’ selection strategy includes key terms for finding the research papers. Rayyan was used as the tool for management, screening, and collection of the literature. In addition to both titles and abstracts that summarize the studies conducted, the full text of the articles was also assessed and reviewed for the eligibility criteria [14]. One reviewer (HETO) screened the titles and abstracts to filter records from PMC NCBI, Scopus, and Cochrane Library for full-text eligibility screening, while all collaborators participated in the full-text eligibility screening to yield final studies to be included in this review. Moreover, the eligibility criteria were followed to strategize the inclusion and exclusion of materials.

### Data extraction

The published studies that passed the screening for eligibility criteria were picked as the chosen papers. These papers were further assessed and analyzed, and the inclusion and exclusion criteria were considered. The data from the chosen studies were extracted using proper study characteristics, such as (1) the title of the research paper or article; (2) the publication year; (3) samples or matrices used for detection of DNA methylation markers; (4) methods of testing; (5) DNA methylation markers detected; and (6) sensitivity and specificity results. Four investigators (AATL, JLAC, ALAA, and CGS) independently extracted data through a standardized form in each included eligible paper. Any conflicts were resolved by another investigator (HETO). Summary of the data extracted is shown in Table 1.

**Table 1.** Master list of CRC DNA methylation markers and their corresponding sample types based on the list of eligible research articles

SI no.	Title, Year	Reference no.	Sample type	Population		Age range (years)	CRC stage		DNA methylation marker
				Male	Female		0–I	II	
1	Identifying potential DNA methylation markers in early-stage CRC, 2020	[10]	FFPE tissue; Blood plasma	37	14	34–77	36	6	SFMBT2, VAV3-AS1, ZNF132, KCNQ5, ITGA4, THBD, FBN1, EVC, C9orf50, TWIST1, ZNF304
2	Blood leukocytes methylation levels analysis indicate methylated plasma test is a promising tool for CRC early detection, 2011	[11]	Blood plasma	55	36	22–89	13	31	SEPT9, SDC2
3	A novel cfDNA methylation-based model improves the early detection of CRC, 2021	[12]	Tissue DNA samples; plasma cfDNA	287	202	18–89	66	86	SEPT9, BCAT1, IKZF1, cg10673833
4	Evaluation of epigenetic methylation biomarkers for the detection of CRC using ddPCR, 2023	[13]	FFPE tissue	57	48	-	24	35	BCAT1, GATA5, IKZF1 (V1), IKZF1 (V2), IRF4, ITGA4, HIC1, NPY, SDC2, SEPT9, WIF1
5	Combined SEPT9 and BMP3 methylation in plasma for CRC early detection and screening in a Brazilian population, 2023	[14]	Blood plasma	11	32	57.1 (mean)	9	9	SEPT9, BMP3
6	CRC detected by liquid biopsy 2 years prior to clinical diagnosis in the HUNT study, 2023	[3]	Blood plasma	32	40	69.8 (mean)	0	11	AGBL4, ALX4, BCAT1, BMP3, FLI1, GRIA4, IKZF1, NDRG4, NPTX2, PRIMA1, RARB, SEPT9, SDC2, SFRP1, SFRP2, SLC8A1, TWIST1, VIM, WNT5A, ZNF331
7	Combining methylated SEPTIN9 and RNF180 plasma markers for diagnosis and early detection of gastric cancer, 2023	[15]	Blood plasma	384	176	<65, >65	94	53	SEPT9, RNF180
8	cfDNA methylation profiles enable early detection of colorectal and gastric cancer, 2016	[16]	Blood plasma	28	42	25–89	10	25	SEPT9, ATXN1, PCDH10, MYO1G, NGFR, IKZF1, ITGA4
9	Circulating-tumor DNA methylation of HAND1 gene: a promising biomarker in early detection of CRC, 2017	[17]	Blood plasma	18	12	31–68	-	9	HAND1, SEPT9

1 cfDNA, cell-free DNA; CRC, colorectal cancer; ddPCR, droplet digital PCR; FFPE, formalin-fixed paraffin-embedded.

### Quality assessment

The quality of the included studies was assessed using the Quality Assessment of Diagnostic Accuracy Studies-2 (QUADAS-2) tool, as recommended by the Healthcare Research and Quality Agency, Cochrane Collaboration, and the U.K. National Institute for Health and Clinical Excellence [15]. The tool evaluates the risk of bias and applicability concerns across 4 domains: patient Selection, Index Test, Reference Standard, and Flow and Timing. All domains are assessed for risk of bias, while only the first 3 domains are additionally evaluated for concerns about the applicability of the findings to the review question. A “No” response to a signaling question was not automatically categorized as a high risk of bias unless deemed critical to the domain’s overall evaluation.

## Results

**Figure 1.**
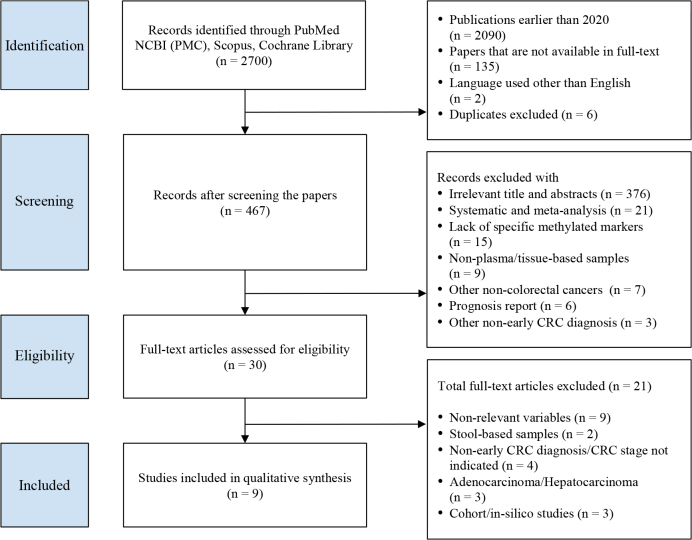
Flow chart of study selection process. CRC, colorectal cancer; PMC, PubMed Center.

A total of 467 articles were identified after excluding publications older than 5 years, those without full-text availability, non-English articles, and duplicates. Screening the review of titles and abstracts yielded 30 studies deemed eligible for fulltext assessment. Of these, 21 were excluded for reasons that apply to the following criteria: irrelevant variables (n = 9); stool-based samples (n = 2); lack of focus on early CRC diagnosis or unspecified CRC stage during sample collection (n = 4); the focus is on adenocarcinoma or hepatocarcinoma (n = 3); and data obtained from cohort or in silico studies (n = 2). Figure 1 illustrates this systematic review’s literature search and study selection process.

### Study characteristics

After filtering and selecting the research articles for this study, 9 articles published from 2020 to 2024 were reviewed. These articles specifically investigated different DNA methylation markers for the early detection of CRC. Numerous studies utilized different samples; the majority used both plasma and tissue, including formalin-fixed paraffin-embedded (FFPE) tissue samples, as specimens. There were other studies that solely focused on using plasma for detecting DNA markers. In addition, cell-free DNA (cfDNA) used from plasma was also frequently used in most studies that allows for the detection of DNA methylation markers to be detected without any invasive tissue biopsy. Notably, Studies 1 and 3 included both FFPE tissue and blood plasma. Study 2 used plasma only as a sample, focusing on methylation levels in blood leukocytes for CRC detection.

**Figure 2.**
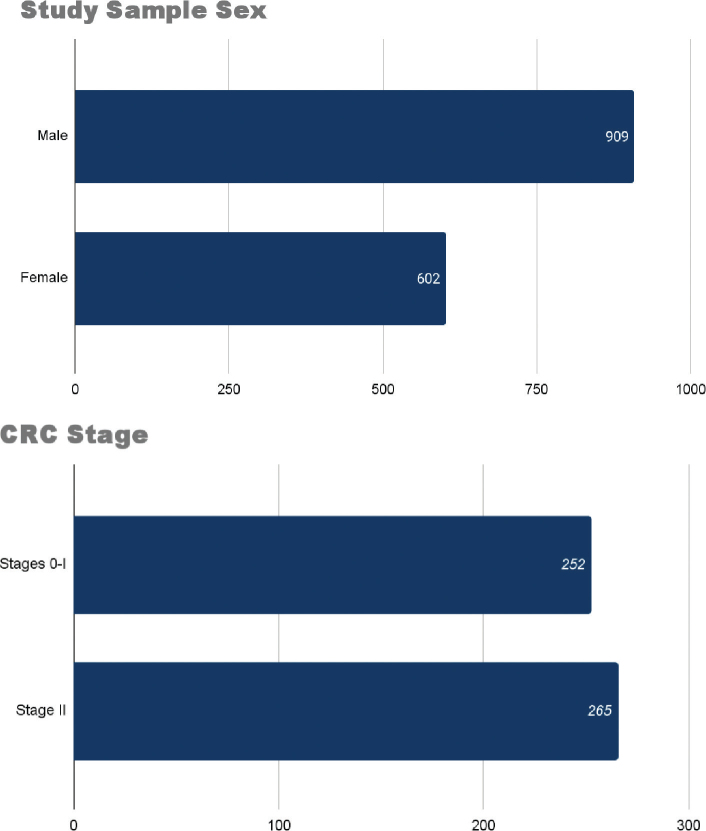
Summary of study samples demographic profile based on sex and early CRC stage. CRC, colorectal cancer.

The sample sizes varied, with the smallest study having 30 participants (Study 9) and the largest study with 560 participants (Study 7). The population in all studies included male and female participants aged ≥18 years, excluding pediatric and adolescent patients. Moreover, the studies focused on a range of CRC stages, with 1 study concentrating on the latest early stage of CRC (Stage II), as seen in Study 9, while the rest included patients with all early stages (Stages 0–II). Figure 2 summates the study samples demographic profile based on sex and early CRC stages.

**Figure 3.**
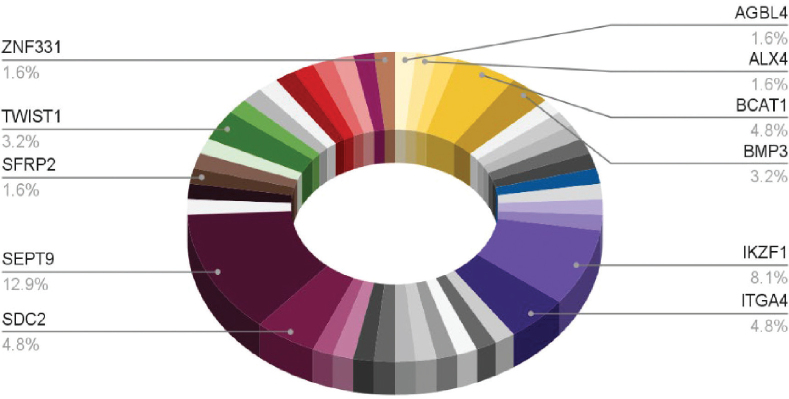
Percentages of identified CRC DNA methylation markers from the list of eligible research articles. CRC, colorectal cancer.

The DNA methylation markers that were assessed were different for every study. Some DNA markers such as SEPT9, SDC2, BCAT1, BMP3, IKZF1, and TWIST1 were studied more frequently as they are potential biomarkers for early detection, screening, and prognosis of CRC (Studies 1, 2, 3, 4, 5, and 7). Moreover, several studies utilized the panels of markers to measure their combined diagnostic effectiveness (Studies 5, 6, 7, and 8). For instance, Study 6 explored a large panel, including markers like AGBL4, ALX4, and BCAT1, while Study 8 looked at markers such as SEPT9, ATXN1, and MYO1G. In addition, Figure 3 presents the percentages of identified CRC DNA methylation markers from the studies.

### Diagnostic performance of plasma-based DNA methylation markers for early CRC detection

Several studies have evaluated the diagnostic effectiveness of plasma-based DNA methylation markers for the initial staging of CRC, focusing on the effectiveness of screening. As shown in Table 1, out of the 9 studies reviewed, 6 studies utilized blood plasma as the sole sample type for DNA methylation marker analysis, while 2 blood plasma was one of the samples used. In the gathered studies, SEPT9 was the most commonly investigated marker, demonstrating sensitivity ranging from 48.2% to 75.8% and specificity between 69% and 94.7%, as observed in Studies 2, 3, 5, 6, 7, 8, and 9. Another promising marker, HAND1, was studied in Study 9, wherein it reached 93.3% sensitivity and 80.0% specificity, which indicates a high diagnostic capacity. However, single markers such as BMP3 examined in Study 5 had low sensitivity between 40.0% and 50.0% and a high specificity of 90.0%. Furthermore, in some cases, more than 1 methylation marker was used, and accuracy tended to be even higher than when using a single marker. For instance, integrating BCAT1/IKZF1, as identified in Study 3, had a sensitivity of 66.0% and specificity of 95.0%. Moreover, the results show that the sensitivity of plasma-based markers was slightly lower than that of tissue-based markers but with excellent specificity, suggesting their continued use as noninvasive diagnostic tools. For example, Study 3, which analyzed SEPT9, shows a sensitivity of 48.2% and a specificity of 91.5%, illustrating the trade-off between ease of collection and diagnostic performance.

### Methodological approaches for identifying CRC DNA methylation markers

Different DNA extraction and isolation methods were carried out among the 9 studies (Table 2). The most used were QIAamp kits (Studies 1 and 5) and MagMAX Cell-Free DNA Isolation Kit (Studies 3 and 6), while some utilized specialized kits, like the AllPrep DNA/RNA FFPE Kit (Study 1). Moreover, methods such as quantitative methylation-specific Polymerase Chain Reaction (qMS-PCR), droplet digital PCR (ddPCR), and bisulfite sequencing were predominantly used for quantifying DNA methylation (Studies 1, 3, and 5). Additionally, realtime PCR (RT-PCR) (Study 1) and methylation-specific PCR (MSP) (Studies 6 and 8) were employed to quantify methylation levels in the respective samples.

**Table 2.** Methods used for the identification of CRC DNA methylation markers

SI No.	DOI	Sample type	Extraction & isolation method	Quantification of methylation method	Markers identified
1	10.1016/j.ygeno.2020.06.007	FFPE tissue; Blood plasma	AllPrep DNA/RNA FFPE Kit (QIAGEN)	RT-PCR	SFMBT2, VAV3-AS1, ZNF132, KCNQ5, ITGA4, THBD, FBN1, EVC, C9orf50, TWIST1, ZNF304
2	10.7150/jca.57114	Blood plasma	QIAamp DNA Blood Mini Kit (QIAGEN)	Bisulfite Sequencing and PCR Assays	SEPT9, SDC2
3	10.1002/1878-0261.12942	Tissue samples; plasma	MagMAX Cell-Free DNA Isolation Kit (Thermo Fisher Scientific)	Bisulfite conversion and targeted sequencing	SEPT9, BCAT1, IKZF1, cg10673833
4	10.1038/s41598-023-35631-5	FFPE tissue	QIAamp DNA Kit (QIAGEN)	ddPCR using Bio-Rad QX200 system	BCAT1, GATA5, IKZF1 (V1), IKZF1 (V2), IRF4, ITGA4, HIC1, NPY, SDC2, SEPT9, WIF1
5	10.1002/cam4.6224	Blood plasma	QIAmp circulating Nucleic Acid Kit (QIAGEN)	ddPCR	SEPT9, BMP3
6	10.1038/s41416-023-02337-4	Blood Plasma	MagMAX Cell-Free DNA Isolation Kit (Thermo Fisher Scientific)	MSP with Zymo Lightning Conversion Reagent	AGBL4, ALX4, BCAT1, BMP3, FLI1, GRIA4, IKZF1, NDRG4, NPTX2, PRIMA1, RARB, SEPT9, SDC2, SFRP1, SFRP2, SLC8A1, TWIST1, VIM, WNT5A, ZNF331
7	10.1002/cac2.12478	Blood plasma	QIAamp Circulating Nucleic Acid Kit (QIAGEN)	qPCR	SEPTIN9, RNF180
8	10.62347/TPTQ3682	Blood plasma	MagMAX Cell-Free DNA Isolation Kit (Thermo Fisher Scientific)	MSP with Zymo Lightning Conversion Reagent	SEPT9, ATXN1, PCDH10, MYO1G, NGFR, IKZF1, ITGA4
9	10.1186/s12920-024-01893-9	Blood plasma	AddPrep Genomic DNA Extraction Kit (Addbio)	qMS-PCR	HAND1, SEPT9

1 CRC, colorectal cancer; ddPCR, droplet digital PCR; FFPE, formalin-fixed paraffin-embedded; mPCR, multiplex PCR; MSP, methylation-specific PCR; qMS-PCR, quantitative methylation-specific PCR; qPCR, quantitative PCR; RT-PCR, real-time PCR.

The studies utilized a variety of samples as shown in Table 2, with the majority using both plasma and tissue, including FFPE tissue samples. Other studies focused solely on using plasma. The use of cfDNA from plasma was particularly frequent, allowing for the non-invasive detection of methylation markers. For instance, the MagMAX Cell-Free DNA Isolation Kit was commonly used to extract cfDNA from plasma samples. However, the AllPrep DNA/RNA FFPE Kit was specifically employed for isolating DNA from solid tissue specimens. This variety in sample types directly influenced the choice of extraction and isolation methods used across the studies.

### Diagnostic performance of tissue-based DNA methylation markers for early CRC detection

Among the 3 tissue-based DNA methylation marker studies, a total of 23 (SFMBT2, VAV3-AS1, ZNF132, KCNQ5, ITGA4, THBD, FBN1, EVC, C9orf50, TWIST1, ZNF304, SEPT9, BCAT1/IKZF1, cg10673833, BCAT1, GATA5, IKZF1 (V1), IKZF1 (V2), IRF2, HIC1, NPY, SDC2, WIF1) markers were analyzed (Table 3), with SEPT9 being involved in Studies 3 and 4, and ITGA4 being involved in Studies 1 and 4. The data generally revealed a sensitivity range of 43.80%–94.10% and a specificity range of 78.10%–100%. For the sensitivity of the methylated markers, SFMBT2, VAV3-AS1, KCNQ5, C9orf50, and ITGA4 (Study 2) performed >90%, while TWIST1, ZNF304, SEPT9, BCAT/IKZF1, GATA5, IKZF1 (V2), HIC1, and WIF1 performed <75%. On the contrary, the specificity of the methylated markers demonstrated high performance, with nearly all markers identified having a specificity >90%, and both TWIST1 and ZNF304 achieving a 100% specificity. SFMBT2 and ITGA4 are both highly sensitive and specific, increasing their reliability and suggesting their potential as robust biomarkers. However, for other markers that exhibit variability, such as ZNF304, SEPT9, BCAT1/INKF1, and WIF1, the importance of considering the specific context or use should be noted, especially when interpreting results. Overall, tissue-based DNA methylation markers leaned toward greater specificity and above-normal sensitivity, meaning a false positive would be less likely to occur.

**Table 3.** Sensitivity (%) and specificity (%) of each identified DNA methylation marker

SI No.	DOI	Sample type	Markers identified	Sensitivity (%)	Specificity (%)
1	10.1016/j.ygeno.2020.06.007	FFPE tissue; Blood plasma	SFMBT2	92.20	94.60
			VAV3-AS1	92.20	89.20
			ZNF132	86.30	94.60
			KCNQ5	90.20	89.20
			ITGA4	90.20	94.60
			THBD	86.30	94.60
			FBN1	86.30	91.90
			EVC	84.30	86.50
			C9orf50	94.10	86.50
			TWIST1	72.60	100.0
			ZNF304	66.70	100.0
2	10.7150/jca.57114	Blood plasma	SEPT9	75.80	94.70
			SDC2	60.40	86.80
3	10.1002/1878-0261.12942	Tissue DNA samples; plasma cfDNA	SEPT9	48.20	91.50
			BCAT1/IKZF1	66.00	95.00
			cg10673833	89.70	86.80
4	10.1038/s41598-023-35631-5	FFPE tissue	BCAT1	73.30–75.20	92.40–94.30
			GATA5	73.30–74.30	91.40–92.40
			IKZF1 (V1)	75.20	93.30
			IKZF1 (V2)	70.50	95.20
			IRF2	81.90–82.90	90.5–91.40
			ITGA4	82.90	88.60–89.50
			HIC1	43.80	78.10
			NPY	80.00	90.50
			SDC2	75.20–76.20	94.30–95.20
			SEPT9	70.50–72.40	88.60–90.50
			WIF1	65.70	96.20
5	10.1002/cam4.6224	Blood plasma	SEPT9	50.00	90.00
			BMP3	40.00	90.00
6	10.1038/s41416-023-02337-4	Blood plasma	AGBL4	40.30	77.50
			ALX4	-	-
			BCAT1	43.10	64.80
			BMP3	41.70	76.10
			FLI1	38.90	76.10
			GRIA4	-	-
			IKZF1	43.10	78.90
			NDRG4	62.50	47.90
			NPTX2	48.60	74.60
			PRIMA1	-	-
			RARB	-	-
			SEPT9	48.60	69.00
			SDC2	41.70	70.40
			SFRP1	40.30	77.50
			SFRP2	31.90	85.90
			SLC8A1	45.80	73.20
			TWIST1	-	-
			VIM	45.80	70.40
			WNT5A	51.40	60.60
			ZNF331	50.00	66.20
7	10.1002/cac2.12478	Blood plasma	SEPT9	40.00	96.00
			RNF180	46.20	87.30
8	10.62347/TPTQ3682	Blood plasma	SEPT9	68.00	80.00
			ATXN1	-	-
			PCDH10	-	-
			MYO1G	-	-
			NGFR	-	-
			IKZF1	-	-
			ITGA4	-	-
9	10.1186/s12920-024-01893-9	Blood plasma	HAND1	93.33	80.00
			SEPT9	66.67	86.67

1 cfDNA, cell-free DNA; FFPE, formalin-fixed paraffin-embedded.

### Quality assessment

**Figure 4.**
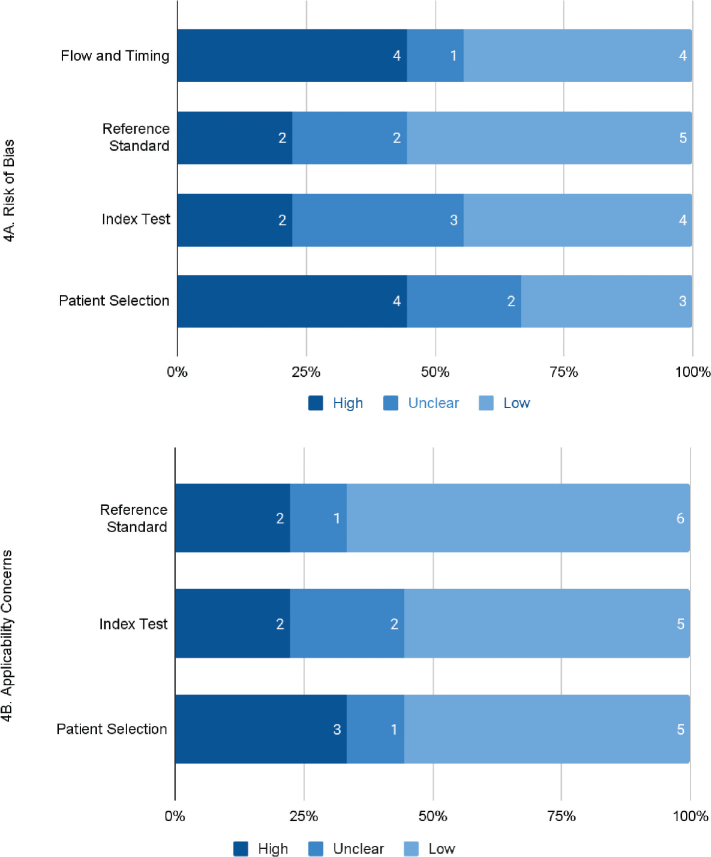
Summary of **(A)** risk of bias and **(B)** applicability concerns of the included papers after QUADAS-2 assessment. QUADAS-2, Quality Assessment of Diagnostic Accuracy Studies-2.

After assessing the 9 eligible studies using the QUADAS-2 tool, considerable results were yielded for review, as shown in the 4 domains of risk of bias and 3 domains of applicability concerns in Figure 4A. The Patient Selection domain remained the most significant concern, with 44% (n = 4) of studies showing a high risk of bias and only 33% (n = 3) showing a low risk, with 22% (n = 2) being unclear. The index test also showed variability, with a notable proportion of studies rated unclear or high risk. Most studies were evaluated as low risk at 56% (n = 5) in the Reference Standard domain, although a small number showed high or unclear ratings. Similar to Patient Selection, Flow and Timing demonstrated a huge bias risk with 44% (n = 4), indicating an inconsistency in how patient progression and timing were handled.

However, applicability concerns were relatively low overall, indicating that the studies’ findings mostly apply to the specific question of early CRC diagnosis in this review. Figure 4B highlights how patient selection induced 56% (n = 5) of studies to exhibit low applicability concerns, while 33% (n = 3) showed high applicability concerns. The Index Test domain had 22% (n = 2) high concerns. The Reference Standard domain showed a high level of credibility, with 67% (n = 6) of studies having low applicability concerns. While some studies answered “No” to questions about consecutive sampling, the overall patient selection process still reflected real-world applicability, leading to a low-risk assessment. The summary of the QUADAS-2 assessment is presented in Table 4, and the graphical representation of bias and applicability concerns is illustrated in Figure 4.

**Table 4.** Quality assessment ratings using the QUADAS-2 scale for reviewed studies (n = 9)

Signaling questions	Patient selection					Index test				Reference standard				Flow and timing		
	Q1	Q2	Q3	Risk of bias	Applicability concerns	Q4	Q5	Risk of bias	Applicability concerns	Q6	Q7	Risk of bias	Applicability concerns	Q8	Q9	Risk of bias
(1) Zhang et al. [16]	Y	N	N	High	Low	N	U	Low	Unclear	U	Y	Unclear	High	Y	N	High
(2) Chen et al. [17]	N	N	Y	Low	High	Y	Y	Unclear	Low	Y	N	Low	Low	N	Y	High
(3) Wu et al. [18]	U	Y	U	Unclear	Low	N	N	High	Low	Y	N	Low	Low	Y	N	Low
(4) Petit et al. [19]	N	N	Y	High	Low	Y	U	Low	High	N	Y	High	Low	N	Y	High
(5) Lima et al. [6]	Y	Y	N	High	High	Y	Y	Low	Low	Y	Y	Low	Unclear	U	Y	Unclear
(6) Brenne et al. [3]	N	Y	U	Low	Unclear	N	Y	Unclear	Low	N	Y	High	Low	Y	U	Low
(7) Nie et al. [20]	U	N	Y	Unclear	Low	N	U	High	Unclear	Y	N	Low	High	N	Y	High
(8) Lei [21]	N	Y	N	Low	High	Y	N	Low	High	Y	U	Low	Low	Y	N	Low
(9) Shavali et al. [22]	Y	N	N	High	Low	N	U	Unclear	Low	U	N	Unclear	Low	Y	N	Low

1 N, no; Y, yes; U, unclear.

1 QUADAS-2, Quality Assessment of Diagnostic Accuracy Studies-2.

## Discussion

The systematic review utilized a total of 9 studies. As shown in Figure 2, these 9 studies included a total population of 909 male and 602 female patients, all of whom had CRC and a total of 1,511 patients combined. The approximate ratio of male to female patients is 60:40, whereas male patients are relatively greater in number than female patients. According to Baraibar et al. [23], it is relatively more prevalent globally among males to have CRC. However, females have higher numbers when it comes to having *BRAF* gene mutation and right-sided cancers. In relation to this, without considering the late stages of CRC, there are a total of 252 patients that are in the earliest stage of CRC (Stage 0–I) and a total of 265 patients that are in the latest early stage of CRC (Stage II) included in the studies. The difference in population between Stage 0–I and Stage II CRC patients has few differences, whereas patients with Stage II CRC are greater by a small margin.

The SEPT9 methylation marker (12.9%) is the most thoroughly researched marker for identifying CRC and can be found in tissue and plasma samples, as highlighted in Figure 3. The identification of SEPT9 in plasma demonstrates a noninvasive option with excellent specificity (69%–96%); nevertheless, its sensitivity (40%–75.80%) differs among plasma studies. Research indicates that enhancing the sensitivity of SEPT9 via plasma necessitates the addition of multi-marker panels, like AKR1B1, to boost diagnostic reliability [25]. Unlike plasma samples, tissue biopsy offers greater sensitivity (70.50%–72.90%) and specificity (88.60%–90.50%) for detecting SEPT9. Jamialahmadi et al. [24] also noted that because of the concentrated tumor DNA found in a tissue biopsy, SEPT9 is established as a reliable tumor marker for CRC. Likewise, IKZF1 is recognized in samples from both tissue and plasma. Plasma samples show reduced sensitivity (43.10%) compared with tissue samples (70.5%–75.2%). Nonetheless, when paired with other markers, like BCAT1, IKZF1 significantly improves diagnostic precision because of the hypermethylation that happens early in oncogenesis and remains from preinvasive lesions to invasive colorectal carcinoma according to Winter et al. [25].

Markers like ITGA4 and SDC2 emphasize the supportive function of tissue and plasma samples in CRC diagnostics. ITGA4 is predominantly identified in tissue samples, showing high specificity and establishing it as a trusted marker for direct access to tumor DNA, guaranteeing accurate detection. Research conducted by Zhang et al. [16] indicates that ITGA4 demonstrates high detection sensitivity for early-stage CRC, rendering it beneficial in clinical environments where precise confirmation of suspected CRC cases is essential. Conversely, SDC2, exhibiting moderate sensitivity and specificity in plasma and tissue samples, is emerging as an encouraging noninvasive biomarker for CRC. Thanks to its efficient functioning in plasma-based tests, SDC2 is identifiable in ctDNA, which makes it valuable for early screening of CRC, particularly in asymptomatic individuals [26].

Table 2 further showcases how the choice of sample type directly influences the selection of isolation and quantification methods for identifying DNA methylation markers. Among the 9 research articles eligible for the criteria, most studies (n = 8) utilized blood plasma samples. However, 3 studies incorporated FFPE tissue specimens (SI no. 1, 3, and 4), with 2 of these studies also employing blood plasma (SI no. 1 and 3). Interestingly, all studies relied on commercial DNA extraction kits for isolating DNA. The most commonly used kit was the QIAGEN QIAamp® DNA Mini Kit (n = 4), followed by the Thermo Fisher Scientific MagMAX™ Cell-Free DNA Isolation Kit (n = 3). A variety of PCR-based techniques were also employed to quantify DNA methylation, including RT-PCR (n = 1), multiplex PCR (n = 1), ddPCR (n = 2), MSP (n = 2), quantitative PCR (qPCR) (n = 1), qMS-PCR (n = 1), and unspecified PCR. Utilization of the QIAamp® DNA Mini Kit in the 9 studies of this review corresponds to the results of the study by Polatoglou et al. [27], which compared the performance of 6 commercial cfDNA kits using human plasma samples and revealed that QIAamp Circulating Nucleic Acid Kit obtained the highest yield and reproducibility. Similarly, an earlier study by Mojtabanezhad Shariatpanahi et al. [28] indicated that QIAamp DNA Blood Mini Kit exhibited a sizebased recovery preference for cfDNA from plasma samples. Larger cfDNA fragments (380 base pairs) were recovered at approximately 27%, while smaller fragments (173 base pairs) exhibited a higher recovery rate of 35%. This only means that smaller cfDNA are more efficiently isolated than larger cfDNA.

In terms of sample types, blood plasma, which contains cfDNA released into circulation, demands methods optimized for low-abundance and fragmented DNA. However, 1 key limitation of using stored plasma as a DNA source is the DNA’s inherent low yield and degradation, regardless of the isolation method employed [29]. This particular challenge is critical in the early detection of on-set stages of CRC, where DNA fragments are present in low-concentration plasma samples. A study by Bhangu et al. [30] highlights the necessity for a sensitive detection method, as the fragmented nature of cfDNA complicates the extraction and isolation techniques of the subsequent methylation analysis. Addressing these limits requires the need for more susceptible downstream quantification methods. According to Malla et al. [4], MSP and ddPCR have shown high effectiveness in handling and analyzing samples. This is rooted in the high sensitivity and capability to identify rare epigenetic changes, as these methods are frequently employed to increase the detection sensitivity. These methods allow absolute quantification and detection of tumor-associated methylation markers in early-stage CRC, even in low-abundance cfDNA samples such as those extracted from plasma [31].

Moreover, FFPE tissue samples, which are incorporated in study SI nos 1, 3, and 4 offer advantages in the stability of DNA. FFPE tissue samples can preserve their morphology and structure, which is why they are widely considered as the gold standard for cancer diagnosis [32]. However, this sample type also presents different limitations due to DNA fragmentation and cross-linking caused by formalin fixation. Lin et al. [33] emphasize that optimized DNA extraction kits such as the QIAamp® DNA Mini Kit help overcome these issues, as this is designed to recover DNA from both plasma and tissue samples. Also, studies that integrate plasma and tissue samples (SI nos 1 and 3) provide a more comprehensive view of the epigenetic landscape of CRC that leads to identifying novel methylation markers for early detection [34]. Generally, sample type, methods of extraction and isolation, and the quantification of techniques play a critical role in early CRC detection.

Blood plasma-based DNA methylation marker performance for early CRC detection was highly variable across the reviewed studies. Markers using HAND1 were evaluated in Study 9, and of all plasma-based markers, HAND1 ranked the highest in sensitivity (93.3%) and specificity (80.0%) and is a promising candidate for CRC detection. Differences in study design and methodology resulted in the variation in sensitivities and specificities of the most extensively studied marker, SEPT9 (Studies 2, 3, 5, 6, 7, 8, 9), as these ranged from 48.2% to 75.8% and 69.0% to 94.7%, respectively. Study 5 also analyzed other markers, such as BMP3, which demonstrated low sensitivity (40.0%–50.0%) but remained with high specificity (90.0%), suggesting its application in specific clinical situations where false negative results may happen and false positives should be limited. Combining markers in Study 3, BCAT1/IKZF1, improved diagnostic performance with a sensitivity of 66.0% and specificity of 95.0%, indicating that the combination of markers provided the most robust diagnostic performance. In Study 6, the marker panel contained AGBL4, ALX4, and BCAT1, but individual sensitivity and specificity metrics were not reported for some, such as ALX4, GRIA4, RARB, and TWIST1, and continued validation is indicated. In Studies 2 and 6, SDC2 showed a sensitivity of 41.75%–60.4% and a specificity of 70.4%–86.8%, indicating moderate diagnostic sensitivity. Also, in Study 6, VIM and WNT5A were performed with limited sensitivity, 45.8% and 51.4%, respectively, and reduced specificity compared with other markers, thus indicating the need for optimization in plasma-based assays. In comparison, tissuebased markers generally yielded higher diagnostic metrics for specificity with above-average sensitivity. Among the 23 methylated markers used, SFMBT2, evaluated in Study 1, had the most clinical potential for detecting the early stages of CRC. This can be supported by its high sensitivity (92.20%) and high specificity (94.60%), constantly being able to maintain a result >90% for both criteria. However, some markers demonstrated incredible performance in 1 metric for sensitivity or specificity. In the case of TWIST1 and ZNF304, both markers exhibited a specificity of 100%, with a sensitivity of 72.60% and 66.70%, respectively. Meanwhile, C9orf50 exhibited a sensitivity of 94.10% and a specificity of 86.50%. This difference in sensitivity and specificity may indicate that some markers should be used based on the context of clinical applications. As such, markers with high sensitivity can identify people with diseases, while markers with high specificity can be used to confirm the disease.

While plasma-based tests like SEPT9 offer a noninvasive and much more patient-friendly alternative, their diagnostic sensitivity is only 48.2%–75.8%. In fact, CMS Decision Memo CAG-00454N (as of 2021) require bloodbased CRC screening tests to achieve at least 74% sensitivity and 90% specificity compared with current gold-standard screenings like colonoscopy [35]. If these benchmarks are not met, such tests risk producing false positives that could unnecessarily alarm patients, drive unneeded invasive procedures like colonoscopies, and even expose individuals to procedural complications. Additionally, this also indicates that plasma-based tests can give false negatives, leading to the failure of early diagnosis. Tissue-based diagnostics, on the other hand, repeatedly give high sensitivity and specificity, but their nature does not make them suitable for widespread screening because of their invasive nature and presence of asymptomatic individuals.

This finding reveals that although the use of plasmabased assays are rising, it still needs improvements to make it reliable for early disease detection, especially if it aims to match the diagnostic accuracy of tissue-based methods while maintaining its noninvasive advantage. Further research and technological progress must develop these tests by adding multiple biomarkers or new detection methods to improve their sensitivity parameters as well as specificity performance for clinical screening and early stage diagnosis. Moreover, it should also be noted that researchers must focus not only on improving test accuracy but also on optimizing costs and clarifying how clinicians should proceed if a test comes back positive.

In the quality assessment of the studies using the QUADAS-2 tool, Figure 4 summarizes the risk of bias and applicability concerns across the 4 domains. The high risk of bias was most prevalent in the “Patient Selection” domain (44%), and Flow and Timing (44%), revealing that most studies had a high risk of bias in patient selection and illustrate potential inconsistency in how patient progression and timing were handled. Thus, this presents a significant limitation of high bias risk in patient selection, potentially affecting generalizability. This predominance of high-risk ratings in patient selection likely stems from retrospective designs of the studies and the lack of consecutive sampling. Additionally, this finding underscores the need for more rigorous patient recruitment strategies in future diagnostic accuracy studies. On the positive side, the Applicability Concern of the Reference Standard and Flow and Timing domains demonstrated a higher proportion of low-risk ratings (67% and 44%, respectively). However, 3 studies focus on non-representative populations, which may affect their broader applicability. Notably, the higher applicability concerns in patient selection reflect limitations in generalizability, particularly for plasma-based assays validated in non-representative populations. Despite this, applicability concerns remained minimal as it shows that the high proportion of low applicability concerns across most domains indicates that the included studies largely align with the review’s objectives of using the correct types of samples and focusing on early CRC diagnosis.

## Conclusion

This systematic review evaluated the diagnostic efficiency of DNA methylation markers derived from plasma and tissue biopsy samples for the early detection of CRC. This paper affirms the diagnostic superiority of DNA methylation markers in tissue-based samples for early CRC detection with their high sensitivity and specificity ensuring accuracy. Thus, this review supports that although tissue-based methods are invasive in sample collection, they remain as the gold standard for the early detection of CRC. Meanwhile, plasma-based markers, such as SDC2, present promising noninvasive alternatives to tissue samples that could significantly enhance accessibility to early CRC screening. However, due to their various displays of reduced sensitivity, they can lead to cases of false negativity in results. Nonetheless, plasma-based samples demonstrate potential, but considerable methodological biases highlight the necessity for more thorough studies to confirm their clinical effectiveness and optimize their diagnostic performance by improving the sensitivity of plasma-based markers to reduce false negative results and therefore, promote widespread screening implementation. Additionally, including more diverse demographics and higher populations improves the generalizability of the findings. Therefore, it is necessary for extensive research to be conducted as plasma-based markers have favorable conditions for early detection of cancer. Also, meeting the 2021 CMS requirement of at least 74% sensitivity and 90% specificity is essential before plasma assays can be widely adopted, as lower specificity risks unnecessary follow-up procedures. In addition to that, addressing methodological limitations (such as patient selection bias and inconsistent timing) will further strengthen future studies. Lastly, incorporating cost-effectiveness analyses and clear clinical management pathways for positive results will help translate these assays into routine practice.
